# Editorial: Networks and graphs in biological data: current methods, opportunities and challenges

**DOI:** 10.3389/fbinf.2025.1685992

**Published:** 2025-09-02

**Authors:** Derek L. Thompson, Hsiang-Yun Wu, Christopher W. Bartlett, William C. Ray

**Affiliations:** 1 The Ohio State University, Columbus, OH, United States; 2 Department of Media and Digital Technologies, Research Institute of Creative Media/Technologies, St. Pölten University of Applied Sciences, St Pölten, Austria; 3 The Abigail Wexner Research Institute at Nationwide Children’s Hospital, Columbus, OH, United States

**Keywords:** networks, graphs, biology, bioinformatics, visual analytics, challenges

## 1 Introduction

Biological systems are rarely made up of siloed processes. Whether in the interplay of factors influencing gene expression, the balance of consumers and producers in food webs, or the interactions between the parts of an enzyme that modulate its activity, hardly anything in biology acts completely independently. Instead, understanding the interdependencies of a biological network is often critical to understanding the behavior of any of the constituent parts of those networks (Efroni and Cohen, 2002; Ma’ayan, 2017; Wang et al., 2024).

At the intersection of Computer Science and Mathematics, Graph Theory provides a rich literature that explores the formal properties of networks, how to compute upon them and what measures can be derived from them (Euler, 1736; Biggs et al., 1986).

## 2 The challenge

Unfortunately, the crossover from the research on networks to their application in biology has been largely *ad hoc* with minimal consideration of which precise graph formalisms are most apt. One does not need to look further than the application of Markov chain models - which represent sequential-neighbor relationships - to enzyme family classification - which are controlled by 3D-spatial distributions of density and charge - to realize that in biology we often use specific network tools because they are approachable rather than because they are conceptually appropriate (Ray et al., 2014).

As a result, few tools exist for accurate computation or visualization of network data, that utilize many features common in biological networks. Even fewer resources exist to help the bio/life-sciences researcher make optimal use of networks with their data. In this Research Topic, we bring together descriptions of several practices for visualizing and computing on network types present in biological data, as well as new tools that present unique capabilities for this field.

## 3 In this research topic

### 3.1 Network analysis of driver genes in human cancers

In Patil et al. two network-based approaches are demonstrated to identify driver gene patterns among tumor samples from the Pan-Cancer Analysis of Whole Genomes. The first approach used a sequence similarity network (SSN) called the Directed Weighted All Nearest Neighbors (DiWANN) network, a more computationally efficient model than more traditional all-to-all distance matrices, that links each node only to the sequence that is closest to it by edit distance. The time complexity of generating the SSN was reduced by employing DiWANN, and additional refinement and data reduction allowed this analysis to be performed using only about 2,200 nucleotides over the conventional 300,000 nucleotides, yet still produced robust results that identified both exclusive and co-occurring driver genes in specific cancer types. These results were supported by their second approach, a bipartite network analysis that showed specific pairs of genes important in those specific cancer types.

### 3.2 A breast cancer-specific combinational QSAR model development using machine learning and deep learning approaches

Karampuri and Perugu delve into Machine Learning (ML) and Deep Learning (DL) approaches to developing structure-activity relationships between quantitative representations of a molecule’s structure and their biological activity. This Quantitative Structure-Activity Relationship (QSAR) model employs molecular descriptors, such as geometric, topological or physiochemical characteristics, and can be used to predict the biological activity of novel structures, or the potential efficacy of novel combinations of structures in combinatorial chemotherapy. In this work, the authors utilize the ${\text{GDSC}}^{2}$ (Genomics of Drug Sensitivity in Cancer drug Combinations) database to establish combinatorial QSAR models with 11 common regression-based ML and DL algorithms, demonstrating a particularly high prediction performance in several models as measured by ${\text{R}}^{2}$ and RMSE when comparing the QSAR model predictions to actual combinatorial IC50 values for cancer treatments.

### 3.3 A layout framework for genome-wide multiple sequence alignment graphs

While multiple sequence alignments (MSAs) have long been a staple of genomic analysis, they are often limited to visualizing alignment blocks that comprise only fragments of individual genes. Therefore it can be difficult to examine features like duplications or translocation of a sequence with traditional visual approaches like parallel coordinate views or dot plots. Schebera et al. examine genome-wide multiple sequence alignments (gMSAs), and propose a methodology by which to examine longer sequences without breaking them into shorter sequence intervals, thereby preventing the loss of order context, and allowing further investigation into pangenome to pangenome comparisons. Their graph layout framework algorithm is based on the Sugiyama framework, but where that framework was applicable to directed acyclic graphs (DAGs), the gMSA graph is a multi-graph with routing for multiple edges that reduces edge intersections and promotes high readability.

### 3.4 Bayesian networks and imaging-derived phenotypes highlight the role of fat deposition in COVID-19 hospitalization risk

Bayesian networks (BNs) represent probabilistic relationships through DAGs, enabling visualization of complex systems and identifying causality between variables. In their study, Waddell et al. use BN modeling to show the link between MRI-derived deposition of body fat and the likelihood of hospitalization in COVID-19 patients. Besides this significant increase in hospitalization risk, the BN modeling also demonstrated the greatest risk to patients with higher amounts of visceral adipose tissue and liver fat. These results provide a model for potential examinations into other associations between patient factors and potential hospitalization risks using BN modeling.

## 4 Opportunities

We hope that this Research Topic of articles provides a vignette of current practices for complex biological network data types, and introduces new tools uniquely suited for the challenges of biological network data. At the same time, we would like to reiterate that there remains work to be done, to optimally apply network representations and the computational tools that can be used upon them, to biological data. Further, even if the full benefits of today's formal graph theory were applied to biology, there are properties of the interactions present in real biological systems that cannot be well-captured by current graph formalisms. Biology for example, deterministically constructs a form of conditional hypergraph (Bretto, 2013) from paired interactions when it “calculates” the impact of multipoint mutations on enzyme activity (Mohan et al., 2022). Graph theory tools enable us to decompose a hypergraph into its underlying primal (or Gaifman) graph (Kuske and Schweikardt, 2018), but derivation of the hypergraph from its primal graph remains an area of research. This strongly suggests that there is still much that the bio/life-sciences can learn from the world of graph/network theory, and perhaps also that graph theory may find new insights by examining implied networks in biology.
